# SOCS1 Mediates Berberine-Induced Amelioration of Microglial Activated States in N9 Microglia Exposed to *β* Amyloid

**DOI:** 10.1155/2021/9311855

**Published:** 2021-11-05

**Authors:** Qi Guo, Chen Wang, Xiaorong Xue, Bin Hu, He Bao

**Affiliations:** ^1^Department of Pharmacy, Xi'an People's Hospital (Xi'an Fourth Hospital), Xi'an 710004, China; ^2^Department of Pharmacy, The Second Affiliated Hospital of Air Force Medical University, Xi'an 710038, China; ^3^Department of Pharmacy, The Second Affiliated Hospital of Xi'an Jiaotong University, Xi'an 710004, China

## Abstract

Attenuating *β* amyloid- (A*β*-) induced microglial activation is considered to be effective in treating Alzheimer's disease (AD). Berberine (BBR) can reduce microglial activation in A*β*-treated microglial cells; the mechanism, however, is still illusive. Silencing of cytokine signaling factor 1 (SOCS1) is the primary regulator of many cytokines involved in immune reactions, whose upregulation can reverse the activation of microglial cells. Microglia could be activated into two different statuses, classic activated state (M1 state) and alternative activated state (M2 state), and M1 state is harmful, but M2 is beneficial. In the present study, N9 microglial cells were exposed to A*β* to imitate microglial activation in AD. And Western blot and immunocytochemistry were taken to observe inducible nitric oxide synthase (iNOS), Arginase-1 (Arg-1), and SOCS1 expressions, and the enzyme-linked immunosorbent assay (ELISA) was used to measure inflammatory and neurotrophic factor release. Compared with the normal cultured control cells, A*β* exposure markedly increased the level of microglial M1 state markers (*P* < 0.05), including iNOS protein expression, tumor necrosis factor *α* (TNF-*α*), interleukin 1*β* (IL-1*β*), and IL-6 releases, and BBR administration upregulated SOSC1 expression and the level of microglial M2 state markers (*P* < 0.05), such as Arg-1 expression, brain-derived neurotrophic factor (BDNF), and glial cell-derived neurotrophic factor (GDNF) releases, downregulating the SOCS1 expression by using siRNA, however, significantly reversed the BBR-induced effects on microglial M1 and M2 state markers and SOCS1 expression (*P* < 0.05). These findings indicated that BBR can inhibit A*β*-induced microglial activation via modulating the microglial M1/M2 activated state, and SOCS1 mediates the process.

## 1. Introduction

Alzheimer's disease (AD) is one of the most common neurodegenerative disorders, characterized as progressive memory and cognitive dysfunctions, leading to great economic and social burden to the family with AD patients [1–3]. However, unfortunately, there is very limited medicine has been proved to be effective for AD. Therefore, searching for effective methods or therapy for AD is very urgent. Many studies reported that accumulation of *β* amyloid (A*β*) in brain tissue is the key pathological character in the development of AD [4, 5]. And a high level of A*β* in the brain can activate microglial cells and induce neuroinflammation [6, 7]. Additionally, chronic inflammation in brain tissue can result in neuronal injury and even death [8, 9]. For this reason, alleviating A*β*-induced microglial activation is regarded as an effective therapy for AD. Activated microglial cells could be divided into two statuses, classic activated state (M1 state) and alternative activated state (M2 state) [10–12]. As the microglial cells of M1 state can secrete proinflammatory cytokines, including tumor necrosis factor *α* (TNF-*α*), interleukin 1*β* (IL-1*β*), and IL-6, the M1 state is believed to be harmful to the brain; in contrast, the microglia of M2 state can release anti-inflammatory and neurotrophic factors, such as IL-10, brain-derived neurotrophic factor (BDNF), and glial cell-derived neurotrophic factor (GDNF), which is considered to be beneficial [13]. Silencing of cytokine signaling factor 1 (SOCS1) is the primary regulator of many cytokines, mainly expressed in immunocytes, including macrophages in the peripheral tissue and microglial cells of the central nervous system (CNS). And many investigations showed that SOCS1 upregulation could reduce microglial activation and neuroinflammation [14, 15].

Berberine (BBR) is a bioactive substance from *Coptis chinensis*, a traditional Asian herbal medicine [16]. Traditionally, BBR is used to treat bacterial gastroenteritis, and recently, an increasing number of studies indicated that BBR can alleviate A*β*-induced neurotoxicity in vivo and in vitro [17, 18]. These evidences above showed that BBR could be effective in treating AD; the mechanism, however, is still obscure.

In the present study, we used A*β* to activate N9 microglial cells to mimic neuroinflammation of AD and investigated the role of SOCS1 in the BBR-induced anti-inflammatory effects and the potential neuroprotection of BBR.

## 2. Materials and Methods

### 2.1. Cells and Reagents

The N9 microglial cells were obtained from the Air Force Medical University. Berberine, *β* amyloid 1-42 peptide (A*β*), Iscove's modified Dulbecco's medium (IMDM), fetal bovine serum (FBS), 3-(4,5-dimethyl-2-thiazolyl)-2,5-diphenyl tetrazolium bromide (MTT), and dimethyl sulfoxide (DMSO) were purchased from Sigma-Aldrich (St. Louis, MO, USA). The enzyme-linked immunosorbent assay (ELISA) reagent kits, including TNF-*α*, IL-1*β*, BDNF, GDNF, and IL-10, were purchased from PeproTech Inc. (USA). The anti-iNOS (ab115819), anti-Arginase-1 (anti-Arg-1, ab124917), and anti-SOCS1 (ab9870) primary antibodies were obtained from Abcam (Cambridge, UK). The Cy-3-labeled secondary antibody, FITC-labeled secondary antibody, and anti-*β*-actin, anti-GAPDH, and secondary horseradish peroxidase-conjugated goat anti-rabbit primary antibody were obtained from Beijing Bioscience Co. Ltd. (Beijing, China). The 4′,6-diamidino-2-phenylindole (DAPI) staining solution was purchased from Beyotime (China).

### 2.2. Cell Culture and Treatments

The N9 microglial cells were cultured in the IMDM medium, containing 5% FBS, 1% (*v* : *v*) penicillin/streptomycin solution (Solarbio Life Sciences, Beijing, China), and 1 mM glutamine. The humidity of the incubator was 100%, the temperature was 37°C, the atmosphere of the incubator consisted of 95% O_2_ and 5% CO_2_, and the medium was changed every 2-3 days. The cells were passaged 2-3 times per week and used within 10 weeks.

**Figure 1:**
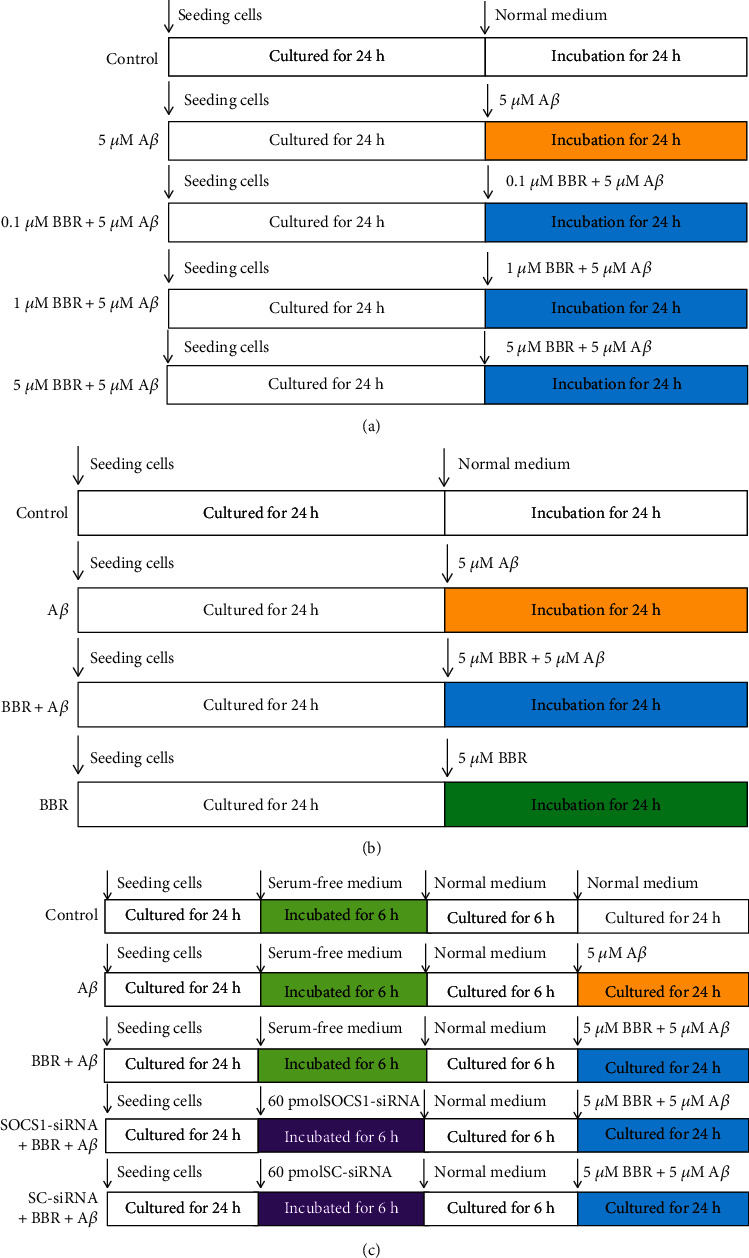
Experimental protocols. (a) Finding a suitable berberine (BBR) concentration. The microglial cells were divided into five groups; after the treatments, tests were performed to find a suitable BBR dose. (b) Investigating BBR-induced effects on proinflammatory factor releases. Cells were divided into four groups; after the treatments, IL-1*β* and IL-6 concentrations were measured. (c) Exploring the role of SOCS1 in the BBR-induced effects on microglial activation. Cells were divided into five groups; after the treatments, microglial M1/M2 activation markers were evaluated to assess microglial activation degree.

To find a suitable BBR treatment concentration, the cells were divided into five groups (Figure 1(a)), including the normal cultured control group, 5 *μ*M A*β* exposure group (A*β*), and three BBR treatment groups (cells were exposed to the medium containing 0.1 *μ*M, 1 *μ*M, or 5 *μ*M BBR plus 5 *μ*M A*β*); after 24 h incubation, iNOS expression and TNF-*α* concentration were measured. To observe BBR-induced effects on proinflammatory factor releases, the cells were divided into four groups (Figure 1(b)), including control, 5 *μ*M A*β* exposure group (A*β*), 5 *μ*M BBR+5 *μ*M A*β* treatment group (BBR+A*β*), and 5 *μ*M BBR treatment group (BBR); after 24 h incubation, IL-1*β* and IL-6 concentrations in the medium were measured. To study the role of SOCS1 in the BBR-induced inhibition of microglial activation (Figure 1(c)), the cells were divided into five groups, including control, 5 *μ*M A*β* exposure group (A*β*), 5 *μ*M BBR+5 *μ*M A*β* treatment group (BBR+A*β*), SOCS1-siRNA treatment group (SOCS-siRNA+BBR+A*β*), and scrambled- (SC-) siRNA treatment group (SC-siRNA+BBR+A*β*); after the treatments, microglial activation markers were evaluated to assess the activation degree of the cells.

### 2.3. Western Blot Analysis

The N9 microglial cells were seeded into a 6-well cell culture plate at a density of 1 × 10^6^ cells/well; after the treatments, the medium was removed and the cells were homogenized on ice with lysis buffer, containing 0.3 M sucrose, 0.15 M NaCl, 2 mM EDTA, 0.3 mM PMSF, 20 mM Tris-HCl, and 10 *μ*g/ml leupeptin. Then, total protein was evaluated by using the Bradford method. And the Western blot analysis was performed as García-Bea et al. previously described [19]. The primary antibodies, including anti-iNOS (1 : 1000), anti-Arg-1 (1 : 1000), anti-SOCS1 (1 : 1000), anti-*β*-actin (1 : 1000), and anti-GAPDH (1 : 1000), were taken in this study. Chemiluminescence technique was used to assess the antigens of the cellular proteins. Computerized analysis software (Bio-Rad Laboratories, USA) was used to perform image analysis.

### 2.4. Enzyme-Linked Immunosorbent Assay

After the treatments, the supernatants of the cell culture plates were collected, and the concentrations of TNF-*α*, IL-1*β*, IL-6, BDNF, GDNF, and IL-10 were measured according to the manufacturers' instructions of the corresponding ELISA kits. The experiments were repeated three times, and the concentrations of the cytokines were expressed as picograms per litre.

### 2.5. Cell Viability Assay

The cells were plated into a 96-well plate at a density of 1 × 10^5^ cells/well; after 24 h incubation, 20 *μ*l MTT solution (5 mg/ml) was added into each well. And after 4 h incubation in the cell incubator at 37°C, the cell medium was removed; then, 150 *μ*l DMSO was added into each well. After 15 min, formazan of each well was dissolved completely. The absorbance of each well was evaluated by using a spectrophotometer at a wavelength of 492 nm.

### 2.6. siRNA Interfering

To downregulate the microglial SOCS1 protein expression, the cells were exposed to 60 pmol SOCS1-siRNA or 60 pmol scrambled- (SC-) siRNA by using the Lipofectamine reagent (Invitrogen, USA) in serum-free medium, under the instructions of the manufacturers. And the cells were incubated for 6 h and then recovered for an extra 6 h before the treatments of the drugs. And the SC-siRNA treatment was considered the negative control.

### 2.7. Immunocytochemistry

N9 microglial cells were seeded into a confocal microscopy-specific cell culture dish at a density of 1 × 10^5^ cells/well. After the treatments, the medium was removed, and the dish was washed three times with phosphate buffer saline (PBS) at room temperature (RT), five min/time. Then, the cells were fixed with 4% paraformaldehyde solution for 30 min; then, the cells were washed with PBS. Next, the cells were blocked with 5% bovine serum albumin (BSA) solution at 4°C after three times washing with PBS. After 1 h BSA blocking, the cells were incubated overnight at 4°C with a primary antibody (iNOS, 1 : 200; Arg-1, 1 : 200). Then, the cells were washed three times with PBS, 5 min/time. The cells were exposed to Cy3-labeled (red) or FITC-labeled (green) secondary antibody for 60 min at RT. At the end of the exposures, 150 *μ*l DAPI solution was added into the dish; after 5 min incubation in the dark, the solution was removed, and the cells were washed three times with PBS, five min/time. Then, the cells were observed, and pictures were taken by using a confocal microscope (FV10i, Olympus, Japan).

### 2.8. Statistical Analysis

SPSS 20.0 for Windows was used to conduct the data of this study. And all data of this study were expressed as means ± Standard Deviation (SD). The results of each group were compared with One-way Analysis of Variance (ANOVA), followed by Tukey's multiple comparisons test. And *P* < 0.05 was considered to be significant.

## 3. Results

### 3.1. Berberine Downregulated iNOS Expression and TNF-*α* Release and Increased SOCS1 Level in Microglia Treated with A*β*

**Figure 2:**
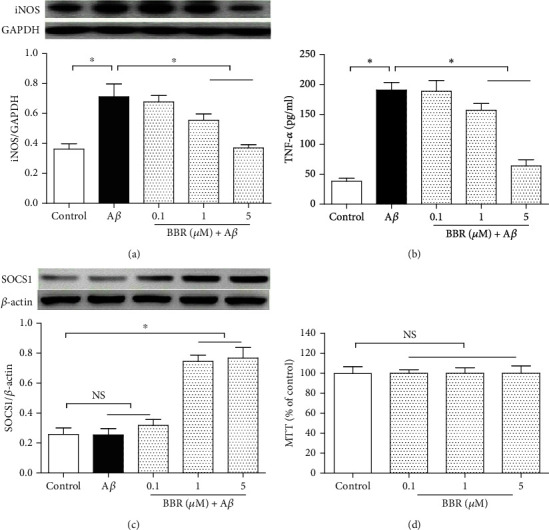
Berberine reduced iNOS expression and TNF-*α* release and upregulated SOCS1 expression in A*β*-treated microglial cells. The N9 microglia were divided into five groups, including the normal cultured control group, 5 *μ*M A*β* group, and three concentrations of berberine treatment groups (medium containing 0.1 *μ*M, 1 *μ*M, or 5 *μ*M berberine plus 5 *μ*M A*β*); after 24 h treatment, iNOS and SOCS1 expressions were measured by using Western blot, and TNF-*α* release was assessed by ELISA. Then, the cells were divided into four groups, including the control and 0.1 *μ*M, 1 *μ*M, and 5 *μ*M berberine treatment groups; after 24 h incubation, the MTT assay was performed to evaluate the cell injury level. (a) Microglial iNOS protein expression (*n* = 4). (b) TNF-*α* concentration in the medium (*n* = 8). (c) SOCS1 protein expression (*n* = 4). (d) Cell viability assay (*n* = 8). Results are expressed as means ± SD. ^∗^*P* < 0.05; NS: no significance.

To explore a suitable dose of BBR, in this experiment, the N9 microglia were divided into the control group, A*β* exposure group (cells were treated with the medium containing 5 *μ*M A*β*), 0.1 *μ*M BBR+5 *μ*M A*β* group (cells were treated with the medium containing 0.1 *μ*M BBR plus 5 *μ*M A*β*), 1 *μ*M BBR+5 *μ*M A*β* group, and 5 *μ*M BBR+5 *μ*M A*β* group; after incubation for 24 h, we took the Western blot analysis to assess iNOS and SOCS1 expression levels, and the TNF-*α* concentration in the medium was measured by using the ELISA (Figures 2(a) and 2(b)). Compared with the normal cultured cells of the control, A*β* exposure increased the iNOS expression and TNF-*α* release (*P* < 0.05) and did not cause marked change of the SOCS1 expression (Figure 2(c)); 1 *μ*M and 5 *μ*M BBR (but not 0.1 *μ*M) significantly decreased the iNOS expression and TNF-*α* release and upregulated the SOCS1 expression (*P* < 0.05), indicating BBR can reverse the A*β*-induced microglial activation and increase SOCS1 expression. BBR of 5 *μ*M was chosen in the next experiments.

In addition, to exclude the potential toxic effect caused by BBR in this study, microglia were divided into four groups (Figure 2(d)), including the normal cultured control and three BBR exposure groups; after a 24 h incubation, we took the MTT assay to check microglial viability. Compared with the control, the three doses of BBR did not induce obvious effects on cell viability (*P* > 0.05), showing that the BBR-induced anti-inflammations in this study were via pharmalogical effects, but not the toxic effects.

### 3.2. Berberine Inhibited Proinflammatory Factor Releases from A*β*-Treated Microglial Cells

**Figure 3:**
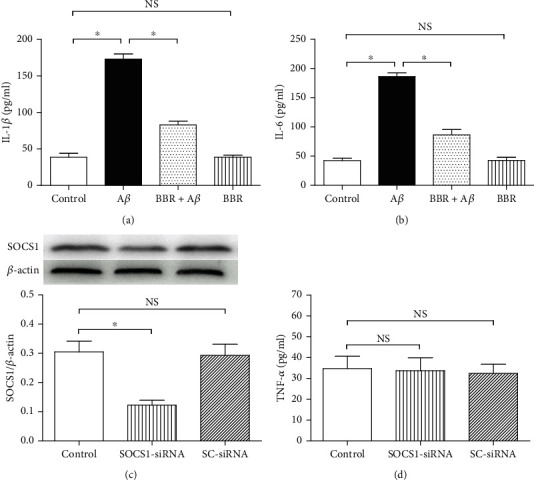
Berberine decreased IL-1*β* and IL-6 releases in A*β*-treated microglia, and SOCS1-siRNA or SC-siRNA did not induce obvious inflammation in untreated microglia. N9 microglial cells were divided into four groups, including the normal cultured control group, 5 *μ*M A*β* exposure group, BBR treatment group (medium containing 5 *μ*M BBR and 5 *μ*M A*β*), and 5 *μ*M BBR treatment group. After 24 h treatments, ELISA was taken to assess IL-1*β* and IL-6 concentrations in the medium. Then, the cells were divided into three groups, including control, SOCS1-siRNA, and SC-siRNA groups; after 6 h incubation, Western blot and ELISA were taken to evaluate the SOCS1 expression and TNF-*α* release. (a) IL-1*β* concentration in the medium (*n* = 8). (b) IL-6 concentration in the medium (*n* = 8). (c) SOCS1 expression level (*n* = 4). (d) The siRNA-induced effects on TNF-*α* release (*n* = 8). Results are expressed as means ± SD. ^∗^*P* < 0.05; NS: no significance.

To assess the proinflammatory factor releases in the presence of BBR, the grouping of microglia is shown in Figures 3(a) and 3(b). After treatment for 24 h, the two proinflammatory cytokines' levels, IL-1*β* and IL-6, were evaluated. Compared with microglia of the normal cultured control, A*β* of 5 *μ*M obviously increased the two cytokines' releases (*P* < 0.05), and 5 *μ*M BBR significantly reversed the A*β*-induced upregulations of the two proinflammatory factors' concentrations (*P* < 0.05), and administration alone with 5 *μ*M BBR did not induce significant changes of the two proinflammatory factors' releases (*P* > 0.05).

To test the inhibitions of SOCS1 expression and cellular inflammation, we took specific siRNA to downregulate the SOCS1 protein expression, and the grouping of the cells was shown in the figures (Figures 3(c) and 3(d)), including the control, SOCS1 gene-specific small interfering RNA (SOCS1-siRNA), and scrambled small interfering RNA (SC-siRNA) groups; after exposure for 6 h, SOCS1 expression and TNF-*α* concentration in the supernatant were assessed. SOCS1-siRNA markedly downregulated the SOCS1 expression, compared with the cells of the control (*P* < 0.05); however, the SC-siRNA did not (*P* > 0.05); meanwhile, either the SOCS1-siRNA or the SC-siRNA did not bring about obvious change of the TNF-*α* concentration (*P* > 0.05). These observations above suggested that the SOCS1-siRNA used in this investigation was effective in downregulating SOCS1 expression without inducing additional inflammation in the cells.

### 3.3. SOCS1-siRNA Blocked BBR-Caused Inhibitions of Microglial iNOS Expression and Proinflammatory Factor Release

**Figure 4:**
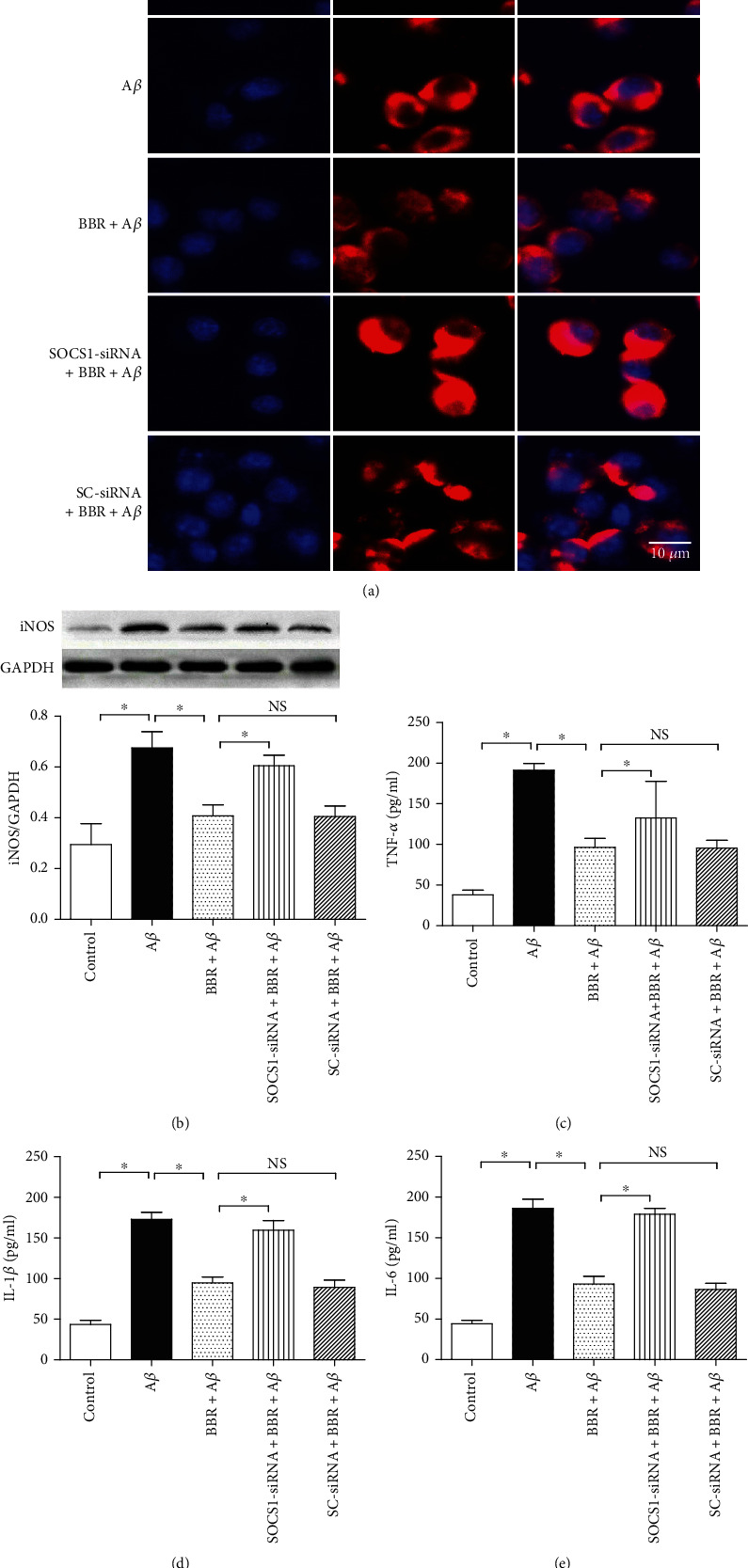
SOCS1-siRNA reversed BBR-induced effects on iNOS expression and proinflammatory factor releases. The cells were divided into five groups, including the normal cultured control group, 5 *μ*M A*β* exposure group, BBR treatment group (medium containing 5 *μ*M BBR and 5 *μ*M A*β*), SOCS1 treatment group (microglial cells were treated with SOCS1-siRNA for 6 h and then exposed to the medium containing 5 *μ*M BBR and 5 *μ*M A*β*), and scrambled- (SC-) siRNA treatment group (microglial cells were treated with SC-siRNA for 6 h and then exposed to the medium containing 5 *μ*M BBR and 5 *μ*M A*β*); then, after 24 h treatment, iNOS expression was observed by using immunocytochemistry staining and Western blot, and inflammatory factors were assessed by using ELISA. (a) Immunocytochemistry staining of microglial iNOS expression. (b) Western blot result of iNOS expression (*n* = 4). (c–e) TNF-*α*, IL-1*β*, and IL-6 concentrations in the medium (*n* = 8). Results are expressed as means ± SD. Bar = 10 *μ*m; ^∗^*P* < 0.05; NS: no significance.

**Figure 5:**
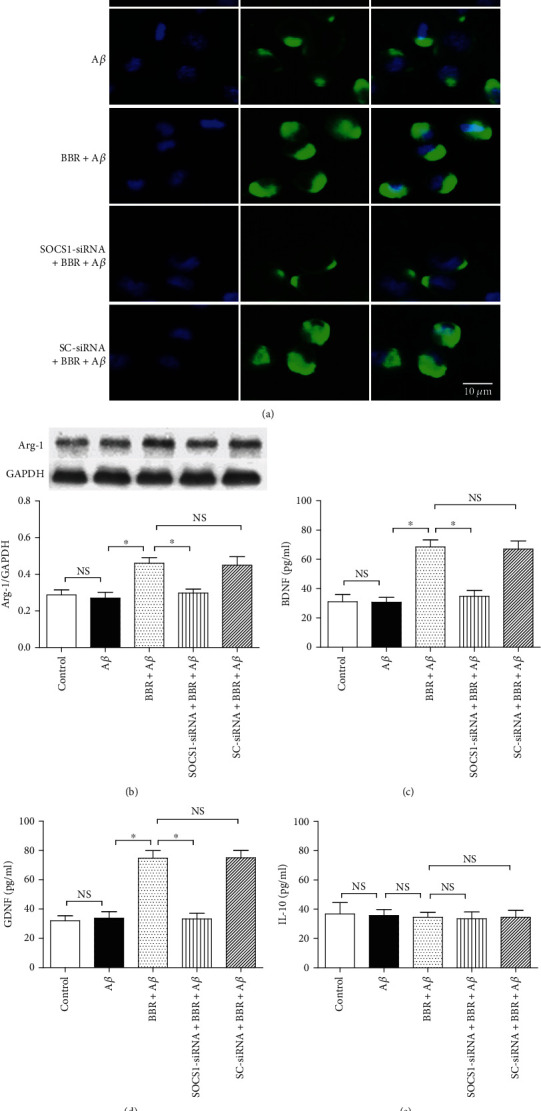
SOCS1-siRNA reversed BBR-induced effects on Arg-1 expression and neurotrophic factor releases. The cells were divided into five groups, including the normal cultured control group, 5 *μ*M A*β* treatment group, BBR treatment group (medium containing 5 *μ*M BBR and 5 *μ*M A*β*), SOCS1 treatment group (microglial cells were treated with SOCS1-siRNA for 6 h and then exposed to the medium containing 5 *μ*M BBR and 5 *μ*M A*β*), and scrambled- (SC-) siRNA treatment group (microglial cells were treated with SC-siRNA for 6 h and then exposed to the medium containing 5 *μ*M BBR and 5 *μ*M A*β*); then, after the treatments, Arg-1 expression was measured by using immunocytochemistry staining and Western blot, and neurotrophic factors were assessed by using ELISA. (a) Immunocytochemistry staining of microglial Arg-1 expression. (b) Western blot result of Arg-1 expression (*n* = 4). (c–e) BDNF, GDNF, and IL-10 concentrations in the medium (*n* = 8). Results are expressed as means ± SD. Bar = 10 *μ*m; ^∗^*P* < 0.05; NS: no significance.

To investigate the role of SOCS1 in A*β*-treated microglial cells exposed to BBR, the SOCS1-siRNA was taken to downregulate SOCS1 protein expression. Compared with the control, 5 *μ*M A*β* significantly increased iNOS expression (Figures 4(a) and 4(b)) and proinflammatory factor releases (*P* < 0.05), such as TNF-*α*, IL-1*β*, and IL-6 (Figures 4(c)–4(e)), and 5 *μ*M BBR obviously inhibited the A*β*-induced upregulations of iNOS and the three inflammatory factors' releases above (*P* < 0.05); however, the SOCS1-siRNA markedly reversed the BBR-induced effects on the iNOS expression and the three cytokines' releases (*P* < 0.05); the SC-siRNA, however, did not (*P* > 0.05). These results above showed that BBR can decrease neuroinflammation, and microglial cell number of M1 activated status in A*β*-treated cells, and SOCS1 might mediate the effects.

### 3.4. SOCS1-siRNA Inhibited BBR-Caused Upregulations of Microglial Arg-1 Level and Anti-Inflammatory Cytokine Secretion

To explore the BBR-induced modulations in microglial M2 activated state and also investigate SOCS1 in the process, bioactive markers of M2 microglial cells, including Arg-1 level and BDNF, GDNF, and IL-10 releases, were measured. In Figure 5, compared with the control group, 5 *μ*M A*β* did not cause marked changes in Arg-1 expression (Figures 5(a) and 5(b)) and BDNF and GDNF concentrations (Figures 5(c) and 5(d)), and 5 *μ*M BBR markedly increased Arg-1 expression and the above two neurotrophic factor, BDNF and GDNF, secretions (*P* < 0.05); however, SOCS1-siRNA (*P* <0.05), but not the SC-siRNA (*P* > 0.05), obviously blocked the BBR-induced upregulations of Arg-1 expression and BDNF and GDNF releases. Interestingly, the concentrations of IL-10 did not change significantly (*P* > 0.05) in the supernatant, and elevated IL-10 concentration can induce anti-inflammatory effect. These results showed that BBR can increase neurotrophic factor releases and the microglial cell number of M2 activated status in A*β*-treated cells, and SOCS1 might mediate the anti-inflammation of BBR.

### 3.5. Berberine Upregulated the SOCS1 Level in N9 Microglia, Which Was Reversed by SOCS1-siRNA

**Figure 6:**
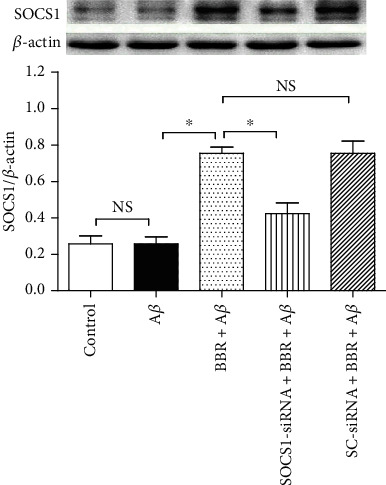
SOCS1-siRNA reversed the BBR-induced effect on SOCS1 protein expression. The cells were divided into five groups, and the treatments were the same as Figure 5, and Western blot was taken to assess SOCS1 protein expression (*n* = 4). Results are expressed as means ± SD. ^∗^*P* < 0.05; NS: no significance.

In Figure 6, compared with the control, 5 *μ*M A*β* treatment for 24 h did not upregulate SOCS1 protein expression (*P* > 0.05), and a dose of 5 *μ*M BBR markedly increased the protein expression of SOCS1; the SOCS1-siRNA, however, obviously inhibited the BBR-induced upregulation of SOCS1 (*P* < 0.05), and SC-siRNA did not cause marked changes of the BBR-induced SOCS1 upregulation (*P* > 0.05).

## 4. Discussion

In this investigation, we observed that treatment with 5 *μ*M A*β* for 24 h increased microglial markers of M1 activated state in N9 microglial cells, including iNOS expression and proinflammatory factors' releases, and the presence of 5 *μ*M BBR reduced the markers' levels of the microglial M1 activated status above, meanwhile upregulating the markers' levels of microglial M2 activated status and the SOCS1 expression, such as Arg-1 expression and two neurotrophic factors' concentrations (BDNF and GDNF) in the medium. Yet, silencing SOCS1 protein expression by using specific siRNA, but not the scrambled siRNA, significantly blocked the BBR-caused changes of biomarkers of microglial M1/M2 status and the SOCS1 expression. These observations showed that BBR inhibits microglial activation caused by A*β* via shifting microglia from M1 to M2 status, and SOCS1 protein might modulate the anti-inflammation of BBR.

AD is an extremely common disorder of the central nervous system, which is age-related and brings about progressive neurological dysfunctions. And it is estimated that about 5 million patients with an age greater than or equal to 65 years are suffering from AD, and by 2050, the total number of AD cases will be at least 13.8 million [20]. Currently, the US Food and Drug Administration (FDA) approved just two medicines, acetylcholinesterase inhibitors and N-methyl-D-aspartate receptor antagonists. However, because of serious side effects and limitations, the two kinds of drugs are rarely prescribed [21, 22]. For this reason, searching for effective medicine or therapy for AD is of great emergency. Cerebral A*β* accumulation is a main biological character of AD, and overdose of A*β* induces neural impairment through two ways. Firstly, overdose of A*β* can injure neuronal cells directly and induces neuron death and neurological disabilities; secondly, overdose of A*β* can stimulate and activate glial cells, including astrocytes and microglial cells; the activated microglia or astrocytes can produce proinflammatory factors and lead to neuroinflammation, and chronic or acute neuroinflammation can also bring about neuronal injury and neurological dysfunctions finally [23, 24]. Therefore, reducing A*β*-induced neuroinflammation is a key step for controlling AD progression. In the present investigation, N9 microglial cells were treated with A*β* to bring about microglial activation, and this cell injury model was used widely in investigating the neuroinflammation of AD [25]. In fact, according to the latest findings of many studies about microglial cells, which could be activated into two different statuses, classic activated state (M1 state) or alternative activated state (M2 state), microglial of M1 status can release a variety of proinflammatory factors and enhance the neuroinflammation; therefore, microglial cells of this state can injure the brain tissue; on the other hand, microglial cells of M2 status could generate a lot of neurotrophic and anti-inflammatory cytokines and reduce the neuroinflammation and increase neural regeneration, so microglial cells of this status are considered to be beneficial [10–12]. For the reasons mentioned above, modulating M1/M2 state or enhancing the microglial M1/M2 state shift is a key method in inhibiting microglial activation-induced neurological disorders.

In China and some other Asian countries, BBR is used universally as a herb for hepatological disorders, microbial infection, and skin infection for many decades [26, 27]. Recently, many investigations indicated that BBR is effective in treating some neurodegenerative diseases. In a scopolamine-induced memory loss model, BBR reduces proinflammatory cytokine release and increases neurotrophic factor generations, including BDNF, GDNF, and cAMP-response element-binding protein (CREB) [28]. In a cell injury model of A*β*-induced BV-2 microglial activation, BBR pretreatment decreased cyclooxygenase-2 (COX-2) and iNOS protein expression levels and also inhibited the productions of monocyte chemotactic protein-1 and IL-6, which are hi-related to inflammatory response [29]. This evidence indicates that BBR can inhibit AD progression by controlling A*β*-induced neuroinflammation. In this investigation, we observed that BBR reduced the markers' levels of microglial M1 state in A*β*-treated N9 microglial cells and also increased the microglial M2 markers' levels in the cells, including BDNF, GDNF, and Arg-1 protein expression, suggesting that BBR can ameliorate the M1/M2 ratio in A*β*-stimulated microglial cells. The findings of our study are similar to the findings mentioned above. However, the exact anti-inflammatory mechanism of BBR in neuronal cells is still not clear. SOCS is not just a protein molecule, which consists of 7 SOCS molecules, from SOCS1 to SOCS7, and the cytokine-inducible SH2 protein (CIS). Being different from the other SOCS molecules, both SOCS1 and SOCS3 could be found in microglia. In addition, many investigations indicated that upregulation of SOCS1 protein can modulate microglial M1/M2 activated state; therefore, we explored the role of SOCS1 rather than the other members of SOCS proteins. And we observed that BBR treatment upregulated SOCS1 expression and inhibited the changes of microglial M1 and M2 states' markers induced by A*β*, yet SOCS1-siRNA obviously blocked the BBR-caused changes of the SOCS1 expression and the microglial M1/M2 state markers, but SOCS1-siRNA or SC-siRNA alone did not induce obvious inflammation in N9 microglial cells, indicating that the BBR-induced anti-inflammation is via inhibiting SOCS1, but not the siRNA molecule. Interestingly, the concentration of IL-10 in the supernatant did not vary significantly in the presence of BBR, indicating that the BBR-induced anti-inflammation is via reducing inflammatory cytokine secretion and elevating neurotrophic factors' releases, but not by enhancing the anti-inflammatory cytokine secretion. And in some other studies, as a main ingredient of *San-Huang-Xie-Xin-Tang*, a formula of Traditional Chinese Medicine, BBR is used in treating cardiovascular and neurodegenerative disorders. BBR can also improve memory and spatial learning ability by increasing A*β* metabolism [30]. Because microglia are the main cells breaking A*β* down in the brain, enhancing A*β* clearance might be another neuroprotective mechanism of BBR in AD. These observations of this study explained, to some extent, the BBR-induced anti-inflammatory mechanism in AD and offered a novel therapeutic target in treating AD. But there are still some limitations to the study. In the first place, the results of this investigation are completely from in vitro experiments, so in vivo experiments and clinical studies are absolutely necessary to test and verify the results of this study. Secondly, we just investigated SOCS1 in the BBR-caused anti-inflammatory effects on A*β*-treated microglia; whether other SOCS proteins are involved in the BBR-caused anti-inflammation is still not clear.

## 5. Conclusions

In summary, in this investigation, we found that BBR decreases microglial activation by improving M1/M2 status in A*β*-treated N9 microglial cells, and SOCS1 protein may mediate the effects.

## Data Availability

The data of this investigation were kept by the corresponding author (He Bao; baohe2yuan@xjtu.edu.cn). If some researchers are in need, they can achieve the data by sending an e-mail to the corresponding author.
